# Complications in the Use of Deepithelialized Free Gingival Graft vs. Connective Tissue Graft: A One-Year Randomized Clinical Trial

**DOI:** 10.3390/ijerph18094504

**Published:** 2021-04-23

**Authors:** Silvestre Ripoll, Ángela Fernández de Velasco-Tarilonte, Beatriz Bullón, Blanca Ríos-Carrasco, Ana Fernández-Palacín

**Affiliations:** 1 Clínica Dental Silvestre Ripoll, Marqués de Paradas 40 Local, 41001 Sevilla, Spain; src@clinicasilvestreripoll.com; 2 Department of Periodontology, School of Dentistry, Universidad de Sevilla, C/Avicena S/N, 41009 Sevilla, Spain; beatrizbullon@hotmail.com (B.B.); brios@us.es (B.R.-C.); 3 Departamento de Ciencias Sociosanitarias, Universidad de Sevilla, 41004 Sevilla, Spain; afp@us.es

**Keywords:** free gingival grafts (FGG), connective tissue grafts (CTG), de-epithelialized free gingival graft (DFGG), late complications, reepithelialization, epithelial bands, cul-de-sac, epithelial cysts, bone exostoses, revascularization

## Abstract

In the treatment of gingival recession, different surgical options have been described: free gingival grafts (FGG), connective tissue Grafts (CTG), and a more recent technique, de-epithelialized free gingival graft (DFGG). They are not procedures exempt from the appearance of complications. Most publications refer to postoperative complications, and there is limited literature regarding the development of late complications (weeks or months). Our working group carried out a study to describe the development of late complications associated with the use of DFGG in comparison with CTG, providing an incidence rate and a classification. Sixty-eight patients with mucogingival problems were selected, and divided into two groups: the Test Group, for which we used DFGG + Coronal Advancement Flap (CAF), and the Control Group (CTG + CAF). All patients were treated at the University of Seville’s dental school to solve mucogingival problems for aesthetic and/or functional reasons. A classification is proposed based on its severity; Major and Minor. Major complications included reepithelialization of the graft, epithelial bands, cul-de-sac, epithelial cysts, and bone exostoses. Minor complications included the graft´s color changes and superficial revascularization. Late major complications were only associated with the use of the DFGG, and the late minor complications developed with the use of the DFGG were much higher than those associated with CTG. CTG appears to be a safer procedure than DFGG in terms of late complications.

## 1. Introduction

Guinard and Caffesse defined a gingival recession as the displacement of the marginal gingival tissue towards the apical junction of the enamel cementum, which develops an exposure of the root surface [1]. As a consequence, the patient may report sensitivity, suffer a higher prevalence of caries, cervical abrasions, and the aesthetic appearance may be compromised [2].

For its treatment, in addition to controlling the causal factors, different surgical options have been described, such as: free gingival grafts (FGG) [3,4], connective tissue grafts (CTG) [4,5], and pedicle flaps [6].

FGG has been used successfully for the augmentation of keratinized tissue and root coverage. However, it is associated with a lower percentage of root coverage compared with other techniques due to its reduced vascularization and its aesthetic appearance as a patch. This is, therefore, its main drawback, leading to it not being used in aesthetic areas [5,7].

Nowadays, a coronally advanced flap (CAF) with subepithelial connective tissue graft (CTG) is considered the gold standard procedure in the treatment of gingival recession-type defects [8]. The combination of CTG + CAF provides a greater vascularization of the graft, achieving a double blood supply, through the supraperiosteal vessels as well as the flap which covers it [9]. Among the benefits obtained, it found higher success rates in terms of complete root coverage, as well as better aesthetic results, as it presents the same color as the pre-existing mucosa compared to the FGG [10,11].

Mucogingival surgery techniques are not free from the occurrence of complications. Early postoperative complications are most commonly described. These develop in a very early state and could lead to bleeding, tooth sensitivity, ecchymosis, and graft necrosis resulting from suture loosening, breakage, or other causes.

Late complications are those that appear within a few weeks or months [12,13,14]. There is limited published literature regarding the development of late complications, and it is mostly related to the use of CTG. Previous studies mainly referred to the formation of cysts [15,16,17,18], the presence of a cul-de-sac [19], bone exostoses formation [20,21], the development of root resorption [22,23,24], and the occurrence of keloids [25,26,27]. Although complications related to the use of the FGG are also reported, most refer to the appearance of cysts [28] and bone exostoses [29,30,31,32].

FGC is a valuable technique; however, some reviews reported disadvantage regarding aesthetic concerns such as pigmentation [33,34]. In addition, some complications have been described as the need for simultaneous augmentation of keratinized gingiva and the requirement of increasing the vestibule depth thereof.

All these late complications refer to the use of CTG or FGG.

More recently, the use of a de-epithelialized free gingival graft (DFGG) has become more widespread. This technique consists of obtaining a graft from a primary free gingival graft that is subsequently deepithelialized outside the mouth to be used as an CTG [20,21].

We did not find any publications referring to the development of late complications specifically associated with the use of DFGG. This is probably due to the fact that it is a more recent and less used technique than the previous ones and therefore has not yet been fully explored in the literature.

After several years of specializing in performing mucogingival surgery techniques using DFGG, our working group at the University of Seville perceived the emergence of some late complications not described in the scientific literature. Among them, we identified the presence of surface reepithelization of DFGG, partial or complete, resulting in a mucous membrane surface similar to the donor area that creates a patch effect, which can be compared to the effect generated in a FGG. However, in these cases, a more irregular morphology depending on the extension area of the reepithelization is present. Sometimes this re-epithelialization is attached by epithelial bands on the graft surface. Moreover, as less relevant complications, our group registered the appearance of multiple superficial blood vessels that alter the aesthetics of the treatment [35].

Conducting meticulously reviews of the publications on free gingival graft and connective tissue graft, we observed a great deficiency in the area of the classification of their complications. We did not find publications that addressed it, either old or recent. Classifications are necessary for the subsequent treatment of complications, and are present in other fields such as wounds, injuries, or surgeries. Our objectives are to describe the late complications associated with the use of the DFGG compared with the CTG, providing an incidence rate, and to provide a classification of complications.

## 2. Materials and Methods

### 2.1. Study Design

Sixty-eight patients were selected for the study, including 39 women and 29 men with a mean age of 39 years (ranging between 19 and 60 years). All patients were treated at the University of Seville´s dental school to solve mucogingival problems for aesthetic and/or functional reasons.

To assess the effect of complications using DFGG, it was compared with the possible drawbacks associated with the gold standard technique in the treatment of recessions (CTG + CAF). For this purpose, we designed a Test group, where we used an DFGG + CAF technique. On the other hand, we created a Control group using the CTG + CAF procedure.

A pilot study was designed to determine the incidence of complications and the sample size needed. Using the N Query Advisor 7.0 program (Statistical Solutions, Cork, Ireland), with a test based on the percentages of complications (between the two groups), we performed a two-tailed test, with a significance of α = 0.05 and a statistical power of 80%. A detection difference of 40% more complications was estimated in the FGG group than in the CTG group (according to a pilot study). The program reported a size of *N* = 28.

We sought to include at least 28 patients in each group, and accounted for the possibility of losing patients due to abandonment or change in residence. Our prospective study increases the numbers from *N* = 28 per group to *N* = 34. Two patients in the Test group dropped out of the study due to change of residence and the final total sample was in the test Group (DFGG + CAF) N = 32 and in the control group *N* = 34.

The assignment of patients to the test group (DFGG + CAF) or control (CTG + CAF) was performed by a balanced randomization using four sizes of blocks that were introduced in closed envelopes. This ensured that the sample sizes in the two groups were the same (well-adjusted), and the envelopes were given as patients arrived.

A statistic descriptive test was performed with frequencies and percentages. For differences in proportions, chi-square contingency and test tables were performed with 95% confidence interval. The Chi Square of independence with continuity correction was used for 2 × 2 tables or Fisher’s exact test for low populated tables.

### 2.2. Inclusion/Exclusion Criteria

Patients were included patients if they had healthy periodontal conditions or successfully treated periodontitis, were nonsmokers or light smokers (<10 cigarettes/day, had an absence of systemic disease, and presented at least one maxillary or mandibular Miller type I/II/III recession [36] that required treatment for aesthetic and/or functional reasons.

On the contrary, patients were excluded with poor oral hygiene, the presence of multiple caries, teeth with active infectious disease, severe malocclusions, or those who had undergone orthognathic surgery, as well as patients who were taking medications that could interfere with the state of periodontal health or the healing of surgical wounds (such as anticonvulsants, immunosuppressants, or Calcium channel blockers) [37].

### 2.3. Surgical Procedure

Informed consent was given by all study participants. Previously, a favorable report from the Ethics and Research Committee of the Junta de Andalucía (Study Code 1259-N-19) was obtained. The patients were all treated at the Seville School of Dentistry (Master of Periodontology and Implants) by one of the main investigators, a well-known professor with years of experience (SR), using microsurgical instruments and 8× magnifying glasses.

Prior to performing the intervention, the operator was informed of the procedure to be performed (DFGG or CTG).

In both groups, the same procedure was carried out to prepare the root surface of the recipient site:

Two releasing incisions were made on the teeth adjacent to the recessions to be treated, which were followed by intrasulcular incisions around the gingival recessions, and a split-full-split thickness flap was raised in the corono-apical direction. The flap was released from the underlying periosteum so that it could be displaced coronally without tension towards the level of the cementum-enamel junction.

**Figure 1:**
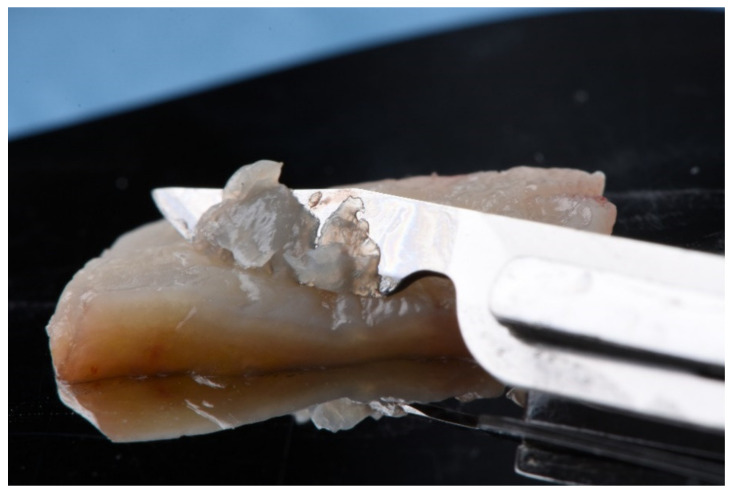
Deepithelialized free gingival graft.

In the donor site, the CTG was obtained from the palatal mucosa using a single horizontal incision in the mesiodistal direction following the technique previously described by Hurzeler [38]. The DFGG was obtained from a previous FGG according to the technique reported by Holbrook and Ochsenbein [39]. It was subsequently de-epithelialized outside the mouth using a scalpel blade (Figure 1). Donor site closure was performed in all cases using continuous suture. In DFGG, a collagen sponge was additionally inserted into the donor site after suturing.

The placement of both DFGG and CTG grafts at the recipient site was carried out following the same procedure. In both cases, the graft was positioned at the recipient site in a manner corresponding to its original orientation, so the DFGG was located leaving its de-epithelialized part facing outwards. Meanwhile the CTG was positioned at the recipient site in a more superficial location facing outwards. Afterwards, a coronal displacement of the flap was performed to cover the graft completely without tension where there was suturing at the interdental level. Finally, the releasing incisions were sutured.

### 2.4. Post-Surgical Instructions

A non-steroidal anti-inflammatory drug was prescribed every eight hours for at least two days to prevent the appearance of pain. Chlorhexidine mouthwash was also prescribed every 12 h for 3 weeks, starting 24 h after surgery. A soft diet and no graft mobilization for at least 3 weeks were indicated. The sutures were removed at 3 weeks in the recipient area.

### 2.5. Index and Classification of Complications

An index of complications was created to speed up the selection task. To this end, a bibliographic review was carried out, making a list of the main complications described in the literature: epithelial cyst, cul-de-sac, bone exostoses, and color change of the grafted area. In addition, we incorporated the main complications not described in the literature, but were observed in our clinical experience: superficial re-epithelialization of the graft, the presence of epithelial bands, and superficial revascularization of the graft.

A definition and description were carried out of each complication including a graphic file.

**Figure 2:**
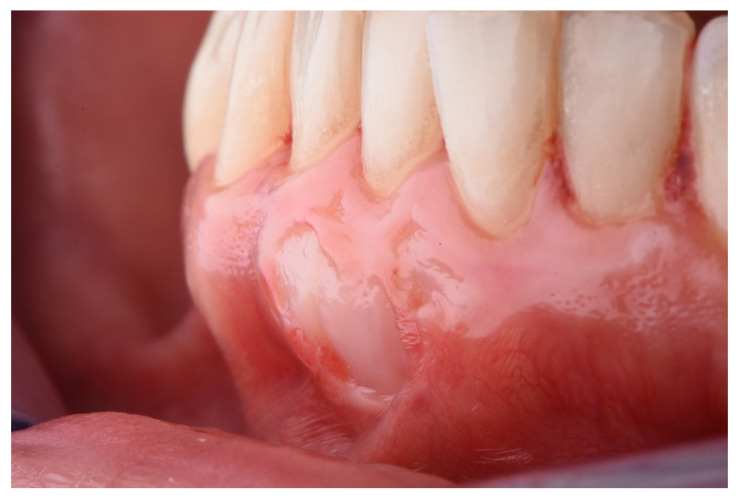
Superficial re-epithelialization.

**Figure 3:**
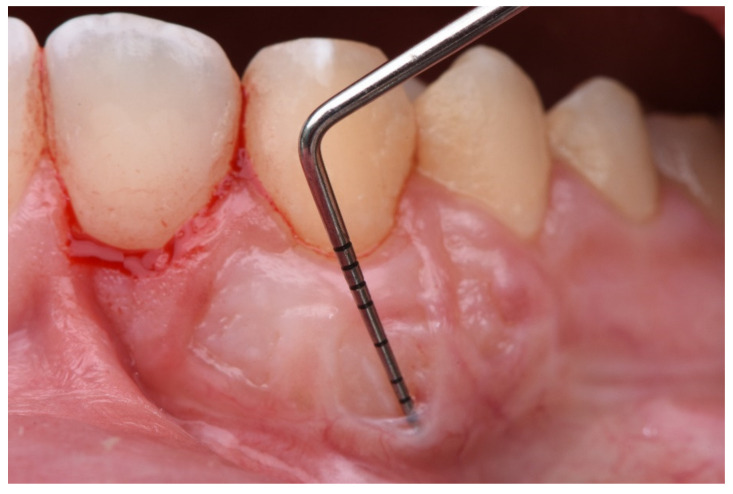
Cul de sac.

**Figure 4:**
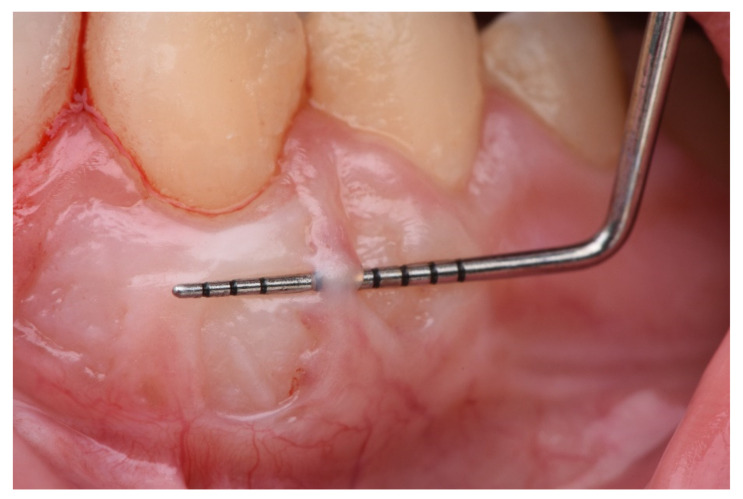
Superficial epithelial bands.

**Figure 5:**
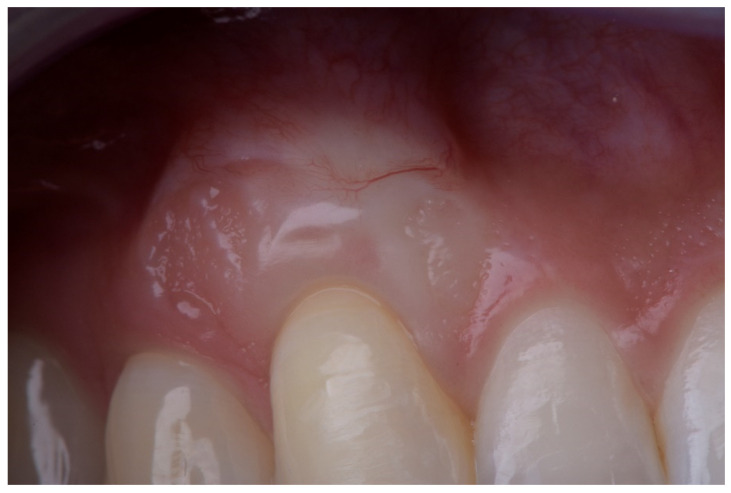
Superficial revascularization.

The description of the following complications was formulated as follows:

1. Epithelial cyst: chronic inflammatory lesion partially or completely delimited by tissue.
2. Cul-de-sac: invagination formation, with probing depths greater than 0.5 mm (Figure 2).
3. Bony exostoses: benign overgrowth of a pre-existing bone.
4. Color change: aesthetic alteration compared with the appearance of the surrounding tissues.
5. Superficial re-epithelialization: proliferation, partial or complete, of the original superficial epithelial layer of the graft, resulting in a mucosal surface similar to the donor area creating a patch effect comparable to the ones produces in an IGL (Figure 3).
6. Superficial epithelial bands: epithelial tissue located on the graft without being adhered to it (Figure 4).
7. Superficial revascularization: proliferation of multiple blood vessels modifying the superficial aesthetics of the graft. (Figure 5).

Complications were classified based on their severity into major and minor. Major complications were considered when additional surgical treatment was required to solve it, or due to their location could cause a considerable alteration of aesthetics. Thus, major complications were considered:

1. The re-epithelialization of the graft; it substantially modifies the aesthetics.
2. The presence of epithelial bands; for being non-adhered retentive areas.
3. The presence of cul-de-sac; retentive and aesthetic effect.
4. The presence of epithelial cysts.
5. Bone exostoses.

Minor complications were considered to be color changes in the grafted area and superficial revascularization.

In this way, we created a graphical guide to complications, with a classification and definition of them, and a graphical database associated with each heading, which we called the Complications Index and which was used to identify and classify the complications that could appear in our study.

### 2.6. Postoperative Controls

Reviews were performed at 7 days, 3 weeks, 2 months, 6 months, and one year.

Photographs were taken at each postoperative review session. All the photographs were reviewed by an independent researcher (A.F.-P.) who did not participate in the surgeries. Their task was to identify and classify the presence of complications. For this purpose, they underwent an initial training with our “Complications Index”. Subsequently, a calibration was carried out to assess their reproducibility when identifying them, performing a Kappa analysis (0.963) after evaluating [10] repeated cases with 48 h of separation.

## 3. Results

To ensure that the groups were analogous, three types of comparison were made: first, by rating whether the recession was unitary or multiple (chi-square test *p* = 0.324); secondly, the location (type) of tooth (chi-square test = 0.353), finally, the type of recession (chi squared test *p* = 0.254). Consequently, results found no significant differences and the groups were comparable.

Complications observed in the Test group (DFGG + CAF) included: superficial re-epithelialization, cul-de-sac formation, appearance of epithelial bands, discoloration changes, and superficial revascularization.

Complications detected in the Control group (CTG + CAF) included: discoloration and superficial revascularization.

We did not find cysts or bone exostoses in either of the two groups.

The statistics relating to the appearance of complications 1 year after the surgery can be seen in Table 1.

### 3.1. Incidence of Major Complications

Reepithelialization of the DFGG was seen in five cases (15.6%) in the Test group (DFGG + CAF) and in no cases in the Control group, with the differences being statistically significant.

The presence of Cul-de-sac was seen in five cases (15.6%) in the Test group (DFGG + CAF) and in no cases in the Control group, with the differences being statistically significant.

The presence of epithelial bands was seen in five cases (15.6%) in the Test group (DFGG + CAF) and in no cases in the Control group, with the differences being statistically significant.

Neither of the two groups experienced cysts or bone exostoses.

### 3.2. Incidence of Minor Complications

Discoloration of the grafted area was observed in 15 cases (46.9%) of the Test group (DFGG + CAF), while in the Control group (CTG + CAF), this was only observed in five cases (14.7%), and the differences were statistically significant.

Superficial graft revascularization was seen in 10 cases (31.3%) in the Test group (DFGG + CAF), while in the Control group (CTG + CAF) it was only seen in two cases (5.9%), and the differences were statistically significant.

## 4. Discussion

Mucogingival surgery procedures, as with any other surgical procedure, are not exempt from the appearance of complications. We must make clear the separation between disadvantages, such as aesthetic results [34] and, complications, such as incomplete healing. Most of the publications report the presence of early postoperative complications (bleeding, pain, inflammation, etc.) [40,41]. Late complications, appear after a few weeks to even months or years of having performed the treatment, are scarce.

As the bibliography indicates, other minimally invasive techniques could be used in the management of the soft tissue, such as laser systems (Er:YAG or Er,Cr:YSGG), according to the expert’s skills, with similar results. [42,43,44]. Some publications suggest that complications can be higher in patients with HIV, and therefore the viral load (CD4/CD8 ratio) must be stable [45].

This prospective study has a double objective of assessing the development and incidence of complications associated with the use of DFGG (Test Group) and comparing this with the development of complications related to the use of CTG (Control Group). Providing a classification and an incidence rate of the most common complications observed after these types of treatments.

### 4.1. Major Complications: Re-Epithelialization, Epithelial Bands, and Cul-de-Sac

The re-epithelialization of the graft was observed in five cases of the Test group, which represents an incidence rate of 15.6%. It is striking that the re-epithelialization of the DFGG has not been described in the literature until now, despite being frequently observed in our clinical practice. Our study considered it the most serious complication, given the appearance of an unsightly patch that it caused. Even when it occurred in areas where aesthetic appearance is less compromised, such as the lower incisors, the aesthetic result was not very acceptable. The re-epithelialization generally did not cover the entire graft, but usually occurred in a localized area and with an irregular shape, leading to patient’s discomfort.

Another surprising finding was locating in these same cases the development of an invagination or cul-de-sac, as well as the presence of epithelial bands on the re-epithelialized graft. In all cases of graft re-epithelialization, the epithelial bands and cul-de-sac appeared to be associated, giving the same incidence rate of 15.6% in the Test group. These complications produced non-adhered retentive areas. To eliminate them, we performed a superficial de-epithelialization with a diamond bur under local anesthesia. The results seem to indicate that patients undergoing an DFGG + CAF procedure are more likely to develop all these complications. It could be suggested that these manifestations may be related. No cases in the control group developed this type of complication. The incidence rate in the Control group was 0% for all the major complications mentioned. These statistically significant differences could be due to the fact that the DFGG could leave epithelial remains that block the adhesion of CAF to the graft area, facilitating the re-epithelialization of the graft, as well as the appearance of epithelial bands and cul-de-sac. Hence, the importance of a correct and thorough de-epithelialization of the graft.

### 4.2. Major Complications: Epithelial Cysts

The appearance of epithelial remains buried under the flap and subsequently generating cysts, manifesting between 9 and 48 months after surgery, has been reported [17,28].

Cysts were one of the major complications described in the literature that we expected to find. Nevertheless, we observed a 0% incidence rate in both the Test and Control groups.

We made three hypotheses in an attempt to explain these results. On the one hand, as the incidence rate of epithelial cysts was so low, our sample size could be insufficient to reveal it. Therefore, it could be necessary to work with a much larger sample. On the other hand, it could be possible that our follow-up time of one year was not enough to reveal this complication, and therefore that could be solved with a longer-term follow-up study.

Finally, it could be possible that the orientation of the graft could influence the formation of this type of complication. In both of our study groups, the graft was sutured in a manner corresponding to its original orientation. The DFGG was sutured with the de-epithelialized face facing outwards, while the CTG was sutured leaving the originally more superficial area facing outwards. This manner of the placement of the graft could be a successful method to avoid cyst formation, since epithelial remains are not trapped underneath.

Notwithstanding, we should take into account that the orientation of the superficial part of the graft towards the outside could also have favored the formation of re-epithelialization and cul-de-sacs in the case of DFGG in the cases that experienced superficial re-epithelialization of the graft. Epithelial cysts could have been produced instead if we had oriented the graft with the de-epithelialized face towards the periosteum at the time of suturing. Although this is just conjecture, it makes sense from a biological point of view. Zuchelli et al., 2009 [27], considered the orientation of the CTG irrelevant at the time of the suture. The author did not refer to the appearance of complications, only focusing in the final result, obtaining the same clinical success in both locations. In addition to this, they did not mention the DFGG. Future research will help us to clarify these questions.

### 4.3. Major Complications: Bone Exostoses

Our studies are not in line with the existing literature on the formation of bone exostoses. These publications describe a series of cases, without providing an incidence rate. Bone exostoses were one of the serious complications described in the literature that we believed could be found in our work; however, we had an 0% incidence rate in both the Test and Control groups.

To explain this event, we present two hypotheses. As in the same case of the epithelial cysts, our sample size could be insufficient to reveal an incidence rate. Furthermore, it could be possible that the follow-up time of 1 year was not sufficient to reveal this complication.

### 4.4. Minor Complications: Discoloration and Superficial Revascularization

Changes in superficial coloration of the grafted area were seen in 46.9% of the cases treated with DFGG + CAF, compared to 14.7% in the Control group (CTG + CAF).

Color change was considered when the color of the grafted area was different from that of the adjacent areas, as described in the Cairo RES index [46]. Cases in which re-epithelialization had occurred were not included. This complication is more a subtle and subjective appreciation, although the analysis carried out by the periodontist in charge of its assessment revealed a consistent reproducibility when detecting these changes.

Although color changes are described in the literature, incidence rates have not been provided. The obvious differences between the two groups are noteworthy, suggesting that the DFGG achieves poorer aesthetic results compared to the CTG. This suggests that perhaps the use of DFGG in aesthetic areas should be considered with caution.

The presence of superficial revascularization on the surface of the grafted area was observed in ten cases in the Test group (31.3%) compared with two cases (5.9%) in the Control group. Generally, there are blood capillaries that rise from the apical to non-advanced coronal in over half of the treated area, and are related to the graft re-vascularization process. This revascularization also produces an unsightly effect.

Due to the postsurgical inflammation of the tissues in the first revision (seven days), the development of complications is not well perceived. Most complications are identified at three weeks after surgery and are maintained in subsequent check-ups at two and six months. In the case of superficial revascularizations, they tend to diminish or even disappear over time.

In our clinical experience, we have observed that this revascularization complication tends to decrease or even disappear over the years, and the tissue tends to harmonize with the rest. It is possible that with a study with a longer observation period, we would have obtained a lower rate of these types of complications. We detected a higher incidence in the group treated with DFGG—therefore, it should be used with caution in aesthetic areas.

## 5. Conclusions

Study results should always be interpreted with caution and multicenter studies with longer-term follow-up are recommended.

A classification of complications according to their severity is proposed; major and minor. Major complications require additional treatment for their correction or significant alteration of aesthetics, and include re-epithelialization of the graft, epithelial bands, cul-de-sac, epithelial cysts, and bone exostoses. In our study, the development of epithelial cysts or bone exostoses was not detected.

Minor complications were considered to be color changes in the grafted area and superficial revascularization.

Regarding the incidence of complications. The presence of late major complications was only associated with the use of DFGG, and the late minor complications that developed with the use of the DFGG were much more common than those associated with CTG. This incident rate may be reviewed in subsequent work.

We can conclude that CTG appears to be a more secure procedure than DFGG in terms of the appearance of late complications. It is much more sensitive to technique, and therefore is not recommended for less experienced clinicians.

## Figures and Tables

**Table 1:** Complications observed in the study. Chi square test for independence, with continuity correction or Fisher’s exact test.

Complications	Control Group (CTG + CAF) (*n* = 32 Patients)	Test Group (DFGG + CAF) (*n* = 34 Patients)	Confidence Interval (95%)
**Major**			
Reepithelialization	0 (0%)	5 (15.6%)	[3.03–28.07%] *p* = 0.023
Cul de Sac	0 (0%)	5 (15.6%)	[3.03–28.07%] *p* = 0.023
Epithelial bands	0 (0%)	5 (15.6%)	[3.03–28.07%] *p* = 0.023
Cysts	0 (0%)	0 (0%)	-
Exostoses	0 (0%)	0 (0%)	-
**Minor**			
Revascularization	2 (5.9%)	10 (31.3%)	[1.259–22.409%] *p* = 0.01
Discoloration	5 (14.7%)	15 (46.9%)	[11.21–53.19%] *p* = 0.007

## Data Availability

Data is contained within the article.
